# Editorial of Special Issue “Nanostructured Light-Emitters”

**DOI:** 10.3390/mi11060601

**Published:** 2020-06-21

**Authors:** Hieu P. T. Nguyen

**Affiliations:** Department of Electrical and Computer Engineering, New Jersey Institute of Technology, Newark, NJ 07102, USA; hieu.p.nguyen@njit.edu; Tel.: +1-973-596-3523

Significant progress has been made in the development of nanophotonic devices and the use of nanostructured materials for optoelectronic devices, including light-emitting diodes (LEDs) and laser diodes, has recently attracted tremendous attention due to the fact of their unique geometry. Nanostructures in small dimensions, comprising nanowires, nanotubes, nanoparticles, etc., can be perfectly integrated into a variety of technological platforms, offering novel physical and chemical properties for high-performance light-emitting devices. Exploring new nanostructured light-emitters and their emerging practical applications is necessary and has attracted considerable investigations from industry and academia. 

This Special Issue contains 3 review papers and 11 research articles, presenting the most recent advances in the device design, simulation, synthesis, characterization and application of such novel nanostructures. The research areas on nanophotonics reported in this issue are broad, covering from ultraviolet (UV) to visible and infrared wavelength regimes. The authors presented their novel results on different types of devices that include inorganic III-V based LEDs, organic LEDs (OLEDs), perovskite LEDs (PeLEDs), and other light emitters/absorbers. 

Wei et al. [1] presented a comprehensive review on β-Ga_2_O_3_ with details in material properties, growth approach, doping, and its application in vertical structure LEDs. Study on β-Ga_2_O_3_ and its function as the substrate for vertical structure LEDs have been intensively investigated. Some of the key advantages of β-Ga_2_O_3_ are high *n*-type conductivity, low lattice mismatch with III-nitrides, and high transparency (>80%) in blue and UV region, enabling β-Ga_2_O_3_ as a highly potential substrates for high-performance light-emitters. 

In another review paper, Zhou et al. [2] summarized recent studies on the interface/surface properties of low-dimensional materials and structures. Importantly, the performance of nanostructured LEDs can be significantly improved by engineering their surface/interface characteristics with a focus on the surface/interface purification, quantum dots-emitting layer, surface ligands, and optimization of device architecture.

The recent progress made in AlGaN nanowires for UV LEDs is reported in the third review paper, entitled “AlGaN nanowires for UV LEDs: Recent progress, challenges, and prospects” [3]. In this paper, Zhao et al. [3] discussed recent developments, the prospects, and the general challenges of AlGaN nanowire UV LEDs. Moreover, AlGaN nanowire UV LEDs on Si, foreign substrates as well as patterned growth approaches with the related device performance are presented.

Enhancing light output power of III-nitride LEDs has been an emerging topic toward the achievement of high efficiency LEDs. In this Special Issue, several approaches have been reported for the enhanced light extraction efficiency (LEE) of III-nitride LEDs. Lei et al. [4] demonstrated that the light output power of the InGaN/GaN LEDs was increased proximately 1.52 times by employing liquid-phase-deposited silver nanoparticle embedded ZnO (LPD-Ag NP/ZnO) thin-film on the top surface of the LEDs. The LPD-Ag NP/ZnO layer served as a window layer in InGaN/GaN LEDs, providing effective surface texture and localized surface plasmon coupling effect for the enhanced light output power of the related InGaN/GaN LEDs. Choi et al. [5]. reported their study on the enhanced photon emission efficiency of AlGaN based UV LEDs by engineering the surface plasmon effect of Pt nanoparticles. They demonstrated that the emission intensity of the surface plasmon-based UV LEDs could be increased 70% higher than that of UV LEDs without using this approach. In another paper, Wang et al. [6] investigated, both theoretically and experimentally, the enhancement in LEE and modulation bandwidth of flip-chip GaN LEDs using photonic crystal. This study shows a promising approach for achieving high-frequency visible light communication. Moreover, Lan et al. [7] theoretically studied the impact of the thin-film flip-chip structure on the enhanced LEE of red/green/blue LEDs. They engineered systematically the impact of the substrate thickness, encapsulation, surface texture, microstructures between the substrate and epilayer, the size, cutting shape, and angle on the performance of the related LEDs.

A novel design of narrow linewidth InGaN/GaN laser diode using distributed Bragg reflector (DBR) is reported in this special issue. Xie et al. [8] demonstrated that the spectral linewidth could be achieved at as narrow was 0.45 nm, using a narrow-band DBR filtering approach.

This Special Issue includes two papers reporting current trends in micro-LEDs (µLEDs) that include nanowire and flip-chip structures. Bui et al. [9] reported that full-color µLEDs with stable emission can be varied from blue to red were successfully demonstrated using nanowire heterostructures grown by molecular beam epitaxy. The µLEDs exhibit excellent optical and electrical properties, showing their promises for the next generation of high-resolution micro-display applications. In another study, Lan et al. reported their study on the LEE of red/green/blue µLEDs using Monte Carlo ray tracing method [7]. Different types of substrates were investigated in this study. They claimed that the LEEs of the µLEDs showed a sharp rise when the chip-size reduced from 30 to 10 µm. This study delivers important approaches for developing high efficiency µLEDs for practical applications.

Studies on OLEDs and PeLEDs are also reported in this Special Issue with two novel structures. Liu et al. [10] studied the effect of charge transport materials on blue OLEDs using a host-dopant system with four different hole transport layers and two different electron transport layers. They reported that the light intensity and lifetime of the OLEDs can be optimized by engineering the charge transport material properties. Moreover, a high brightness emission with a maximum luminance of 3640 cd/m^2^ at 50 mA/cm^2^ was recorded for their blue OLEDs. In another paper, Zhou et al. [11] demonstrated high efficiency PeLEDs using a three-step spin-coated CH_3_NH_3_PbBr_3_ emitter and a PEDOT:PSS/MoO_3_-ammonia composite hole transport layer. They claimed that these approached enable high efficiency PeLEDs that could reach a maximum luminance of 5044 cd/m^2^ and maximum current efficiency of 3.12 cd/A, offering highly promising approach for generating high performance PeLEDs.

Presented in the paper “The Composition-Dependent Photoluminescence Properties of Non-Stoichiometric Zn_x_Ag_y_InS_1.5+x+0.5y_ Nanocrystals”, Feng et al. [12] reported an effective method to synthesize high-quality Zn_x_Ag_y_InS_1.5+x+0.5y_ nanocrystals using a hot facile injection approach. The nanocrystals exhibit tunable photoluminescence from 450–700 nm by varying the Ag composition accordingly. Moreover, this study shows the first investigation of the synthesis of Zn_x_Ag_y_InS_1.5+x+0.5y_ nanocrystal with Zn of 25% and tunable emission wavelength with high quantum yield of 35%. Dang et al. presented a special design of a broadband absorber employing epsilon-near-zero mode with plasmonic metasurfaces [13]. The proposed absorber exhibits a wide range absorption bandwidth of mid-infrared irradiation wavelengths which is perfectly suitable for several applications in detection, sensing, and imaging. 

In the paper “Large-Scale Fabrication of Photonic Nanojet Array via Template-Assisted Self-Assembly”, Zhang et al. [14] demonstrated an advanced manufacturing method to fabricate large-scale homogenized photonic nanojet array with defined pattern and spacing. A template-assisted self-assembly approach was introduced in this study. Importantly, high patterning efficiency of 95% was demonstrated, enabling highly promising applications in super-resolution imaging, subwavelength-resolution nanopatterning, nano objects trapping and detection technology.

We would like to take this opportunity to thank all authors for their valuable contributions to this Special Issue. We would also like to acknowledge all reviewers of this Special Issue for their time and comments.
